# Caffeine ingestion improves specific artistic swimming tasks

**DOI:** 10.1590/1414-431X202010346

**Published:** 2021-02-12

**Authors:** F. Dall'Acqua, G. Cristina-Souza, A.C. Santos-Mariano, R. Bertuzzi, C. Rodacki, A.E. Lima-Silva

**Affiliations:** 1 Grupo de Pesquisa em Performance Humana, Departamento Acadêmico de Educação Física, Universidade Tecnológica Federal do Paraná, Curitiba, PR, Brasil; 2 Grupo de Pesquisa em Performance Humana, Departamento de Educação Física, Universidade Federal do Paraná, Curitiba, PR, Brasil; 3 Grupo de Pesquisa em Nutrição e Exercício, Academia do Campus de Passos, Universidade Estadual de Minas Gerais, Passos, MG, Brasil; 4 Grupo de Estudos em Desempenho Aeróbio, Departamento de Esporte, Escola de Educação Física e Esporte, Universidade de São Paulo, São Paulo, SP, Brasil

**Keywords:** Flexibility, Strength, Power, Muscular Endurance, Performance

## Abstract

The main movements of artistic swimming demand various physical capacities such as flexibility, strength, power, and muscular endurance. The use of ergogenic resources to potentialize performance in this sport, however, is underexplored and deserves investigation. In the present study, we tested whether caffeine ingestion would improve the execution of movements that are essential in a typical figure competition or routines in artistic swimming (i.e., amplitude in the Ariana, height in the Boost and Barracuda, and time maintained in the Stationary Scull techniques). Sixteen experienced female athlete artistic swimmers (17.4±3.2 years of age, 5.6±2.8 years of artistic swimming practice) performed several movements of artistic swimming after having ingested a capsule containing caffeine (5 mg/kg body mass) or cellulose (placebo). Compared to the placebo, caffeine improved latero-lateral amplitude during the Ariana (P=0.035), the height of the Boost and Barracuda (P=0.028 and 0.009), and maintained duration in Stationary Sculling (P=0.012). Bayes factor analysis, however, indicated substantial evidence of a positive effect of caffeine only on the Barracuda and Stationary Scull techniques. These findings indicated that caffeine improved performance during specific artistic swimming movements. Coaches and athletes should consider caffeine ingestion in their supplementation plans.

## Introduction

Synchronized swimming (referred to as artistic swimming since 2017 by the International Swimming Federation) is an Olympic sport that combines swimming, dancing, and gymnastics (1). Competitions are performed as solos, duets, or teams (2), in which the athletes perform artistic routines of elaborate movements in the water accompanied by music (3). A typical routine takes between two to four minutes and is composed of exercises demanding high levels of esthetic, technical, and physical components (2). There is also a “figure competition”, in which the athletes perform a set of mandatory or optional figures, and they are judged regarding the accuracy of positions, transitions, control, extension, speed, height, stability, and uniformity of motion (2). The capability to perform these exercises with excellence requires great flexibility, strength, power, and muscular endurance (4,5).

There are a couple of exercises that are essential in a typical figure competition or routines. For example, the Ariana, a compulsory figure for athletes who are between 13 and 15 years old, requires high levels of flexibility of the lower limbs (2). During Ariana, a back walkover is executed to a split position and then, maintaining the relative position of the legs, the hips rotate 180° and a front walkout is executed (2). Thrusts or propulsion, also called body-jumps, are also among the most recognized compulsory figures of artistic swimming demanding strength and power (2). The most widely used thrusts are the Boost and the Barracuda. In the Boost, athletes must rise headfirst from the water and lift their arms above their heads before the upward momentum of the jump is lost (6). In the Barracuda, athletes must reach the highest upside-down vertical position in which the legs emerge first from the water (6). Artistic swimming also demands hand movements used to propel the body, the so-called “Sculls” (2). One of the most commonly used is the “Stationary Scull”, which is performed by holding the upper arms against the sides of the body with forearms and hands positioned at 90-degree angles to the body. The forearms are then moved back and forth while maintaining right angles to the body, with the resulting pressure against the hands allowing the swimmers to hold their legs above water. The athletes need to maintain Stationary Scull for long periods, demanding high muscular endurance.

These movements demand the combination of various physical capacities, and it is surprising that the use of ergogenic resources to potentialize performance in this sport is underexplored. Due to its ergogenic proprieties, caffeine could be a potential substance to optimize performance during the execution of the main movements of artistic swimming. The majority of studies that explored the effects of caffeine used laboratory-based tests for evaluating strength, power, and muscular endurance (e.g., Wingate test, jumping tests) (7). In contrast, less focus is devoted to more complex tasks demanding strength, power, and muscular endurance such as those involved in the main movements of artistic swimming.

It has been well described in the literature that caffeine ingestion has both central and peripheral effects (8,9). Caffeine acts centrally as an antagonist of the adenosine receptors, preventing a decrease in neuronal activity and muscle recruitment (10). The peripheral action of caffeine is less evident, but it has been associated to an improved muscle calcium release, increasing muscle force (10,11). These central and peripheral effects of caffeine explain the reported improvement in strength, power, and muscular endurance after caffeine ingestion (12–14). In addition, caffeine might act on peripheral and supraspinal sites, blocking pain transmission (15–18). Due to its analgesic proprieties, caffeine ingestion may also have an impact on flexibility (19). Therefore, it is reasonable to hypothesize that caffeine might be able to improve performance during the execution of the main movements involved in artistic swimming. The confirmation of this hypothesis would be useful when determining supplementation plans for artistic swimmers.

Thus, the aim of this study was to investigate whether caffeine ingestion would improve the execution of the main movements involved in artistic swimming. We chose to assess movements involving flexibility (Ariana), strength and power (Boost and Barracuda), and muscular endurance (Stationary Scull). We hypothesized that caffeine ingestion would improve performance during these tasks.

## Material and Methods

### Participants

The required sample size was calculated using G-Power software (version 3.1.7, Heinrich-Heine-Universität Düsseldorf, Germany). Because we were unaware of any previous study that investigated the effects of caffeine on the main movements involved in artistic swimming, the required sample size was calculated using an expected moderate effect size (ES=0.8) of caffeine on strength, power, and muscular endurance (20–23). With an alpha of 0.05 and a desired power of 0.80, the sample size necessary to achieve statistical significance was estimated to be 15 participants. Thus, sixteen experienced female artistic swimmers (5.6±2.8 years of practice, 17.4±3.2 years of age, 54.6±6.4 kg, and 162.2±4.0 cm) participated in this study. Participants were selected based on the following inclusion criteria: 1) had been regularly engaged in an artistic swimming training program for at least four years before the study; 2) had been competing at a national and/or international level in the previous two years; 3) had reached puberty (pubic hair ≥stage 3); 4) had a regular menstrual cycle (27 to 31 days); and 5) had been free from menstrual disorders, such as dysmenorrhea or amenorrhea for the six previous months. Participants were excluded if they reported: 1) any type of muscle injury within the previous six months; 2) use of any medication within the previous month; and 3) use of oral contraceptive pills within the previous three months. Before starting the procedures, the athletes were informed of the requirements, benefits, and risks of the study and signed a consent form, with their respective guardians' permission, when needed. The study was conducted according to the Declaration of Helsinki and was approved by the Research Ethics Committee of the Technological Federal University of Parana (Brazil).

### Design

In the first visit, body mass and height were measured. Although participants were already familiar with the movements used in the tests, they were asked to participate in an initial familiarization session with the experimental procedures. Participants practiced all movements within one hour, exactly as they would be performed during the experimental trials. In the second and third visits, participants performed the tests one hour after they had ingested 5 mg/kg body mass of caffeine or placebo. This caffeine dose was selected because it has been reported to be the most used dose to increase muscle strength and power (24). The second and third visits were performed with a 1-week period between them for washout, adopting a counterbalanced order and crossover design. The second and third visits were performed between 15 to 25 days after menstruation (luteal phase) to avoid any influence of the menstrual cycle on exercise performance (25,26). Athletes were tested one at a time and neither athletes nor researchers were aware of which supplement was being ingested (double-blind design). A list containing all foods and beverages with caffeine was given to each participant. Participants were instructed not to consume foods and beverages contained on the list or alcoholic beverages during the 24 h before each trial. A 24-h period of caffeine abstinence has been reported to fully clear plasma caffeine (27). Participants were also instructed not to perform strenuous exercise in the last 24 h before trials. All trials were performed in the morning (from 8 to 11 AM) to avoid any effect of a circadian rhythm, with the athletes taking their last meal three hours before the start of the supplementation protocol. The athletes were instructed to record their diet 24 h before each experimental session.

### Experimental sessions

The experimental sessions were performed with the athletes wearing a two-piece bathing suit. Anatomic points (lateral and front toes, lateral and medial malleolus of both ankles, medial and lateral epicondyles and patella of both knees, half distance between the patella and the greater femoral trochanter of both thighs, and wrist) were marked with indelible marker for kinematics 2D motion analysis. The indelible mark was maintained on the skin until the next visit to ensure identical localization of the mark and the reproducibility and reliability of the measurements.

**Figure 1:**
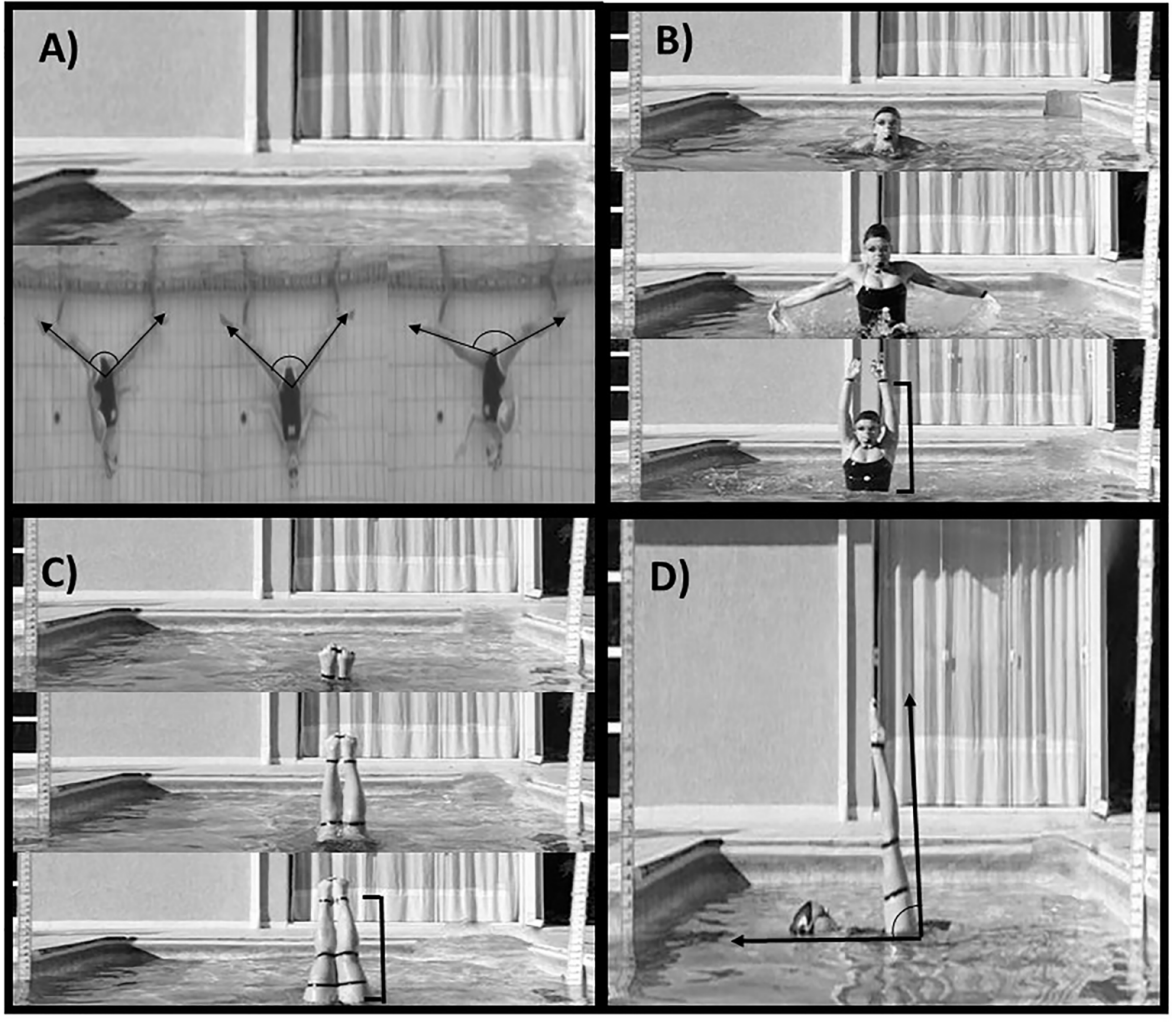
Artistic swimming figures used in the experiment. **A**: Ariana; **B**: Boost; **C**: Barracuda; **D**: Stationary Scull.

Participants ingested a gelatin capsule containing either caffeine (5 mg/kg of body mass) or cellulose (placebo), which had the same color, odor, and shape, with 200 mL of water. They then rested for 40 min and then performed a 20-min warm-up out of the water composed of stretching exercises of both upper and lower limbs (the same warm-up schedule used in their training routine). After the warm-up, participants entered a 2.10-meter deep swimming pool and performed the Ariana figure (Figure 1A) three times (15 s apart), as described in the International Swimming Federation manual (2). Athletes started with the body floating flat on the water, and then a back walkover was executed to a split position, maintaining the relative position of the legs on the surface. The hips than rotated 180° and a front walkout was executed. The second test was a Boost figure (Figure 1B), performed three times (15 s apart) in accordance with Peric et al. (6). Briefly, the Boost started from a vertical swimming position with the participants rapidly raising headfirst from the water and lifting their arms to an upright position. The third test was a Barracuda figure (Figure 1C), performed three times (15 s apart) in accordance with Peric et al. (6). Briefly, the Barracuda started from a back-layout position, and then the legs were raised to vertical position as the body was submerged to a back-pike position with the toes just under the surface, followed by a vertical upward movement of the legs and hips as the body unrolls to obtain a maximal vertical position. The last test was a Stationary Scull (Figure 1D). Athletes assumed a horizontal position with one leg in a vertical position. They were instructed to maintain that position as long they as could by moving their forearms back and forth. The Stationary Scull was interrupted when: 1) the participant was not able to maintain the correct position (thigh angle set at 90° in relation to the water surface); 2) if their patella was emerged surpassing water surface or; 3) if they disengaged from the task (exhaustion). An experienced coach, who did not know which supplement had been ingested, monitored the execution of the movement and gave feedback to return for the correct position when necessary. Criteria 1 and 2 for test interruption were accepted when the athlete was unable to return to the right position after two notifications from the coach. The time supported until task failure was recorded and further used in the analysis.

### Analysis

The Ariana, Boost, and Barracuda positions were recorded using a digital camera Casio Exilim EX-FH20 (Casio Computer CO., Japan) positioned 3 m in front of the athletes, with image acquisition frequency of 200 Hz. The 2D calibration was carried out by two vertical metal stems (with visible marks every 10 mm), which were fixed from the pool floor up to 180 cm above water level and positioned in the filming field with a 2-m distance between them. For the Ariana, the angles between the legs in the frontal (lateral split) and sagittal (anterior-posterior split) planes were measured considering the inner side of the legs (Figure 1A). For the Boost, the maximal vertical distance between the water surface and the wrist immediately before ending the upward momentum was calculated (Figure 1B), while for the Barracuda the maximal vertical distance between the water surface and the ankle was calculated (Figure 1C). All these measures were performed using an image of 2D software analysis (Kinovea^®^, version 0.8.20, USA). The same blind investigator performed all video analyses. To measure the Ariana's amplitudes, the investigator selected the frame in which maximal amplitudes were noted (Figure 1A). To measure the Boost height, the investigator selected the frame in which the wrist reached the maximal height and subtracted from the water surface height (i.e., 2.10 m, Figure 1B). The same procedure was used for the Barracuda, using the frame in which the ankle reached the maximal height (Figure 1C). The average score of the three attempts for each trial was used in further analysis. The same investigator also performed the same analysis twice in a subsample (12 for placebo and 12 for caffeine). In the placebo arm of the study, the mean intra-evaluator coefficients of variation were 0.43±0.72% for right anteroposterior split of the Ariana, 0.75±1.12 % for the left anteroposterior split of the Ariana, 0.34±0.39% to latero-lateral split of the Ariana, 2.74±1.69% for the Boost, and 4.74±3.39% for the Barracuda. The corresponding values for the caffeine arm were 0.32±0.39%, 0.55±0.59%, 0.65±0.60%, 2.47±1.40%, and 4.88±3.65%.

### Statistical analysis

Data were tested for normality using the Shapiro-Wilk test. Once normality was confirmed, the paired *t*-test was used to compare performance between caffeine and placebo conditions. Hedges' g effect size (ES) and 95% confidence interval (95%CI) were calculated using an online calculator (https://effect-size-calculator.herokuapp.com/) from means and pooled standard deviations (SD) to verify the magnitude of the effect of caffeine on performance, assuming values of 0.2, 0.6, 1.2, 2.0, 4.0, and >4.0 as trivial, small, moderate, large, very large, and extremely large, respectively (28). In addition, Bayes factor was also calculated using an online calculator (http://pcl.missouri.edu/bayesfactor) from sample size, *t*-value, and r value on effect size to determine the probability/chance of performance enhancement/impairment in each experimental group. The Bayes factor was interpreted as previously recommended (29,30). To assess whether there was an order effect, a paired *t*-test was used to compare performance between the first and the second trials. Statistical analyses were carried out using SPSS for Windows (20.0.0, Standard Version, IBM, USA). Statistical significance was considered to be P<0.05. Data are reported as mean±SD.

## Results

### Ariana

**Figure 2:**
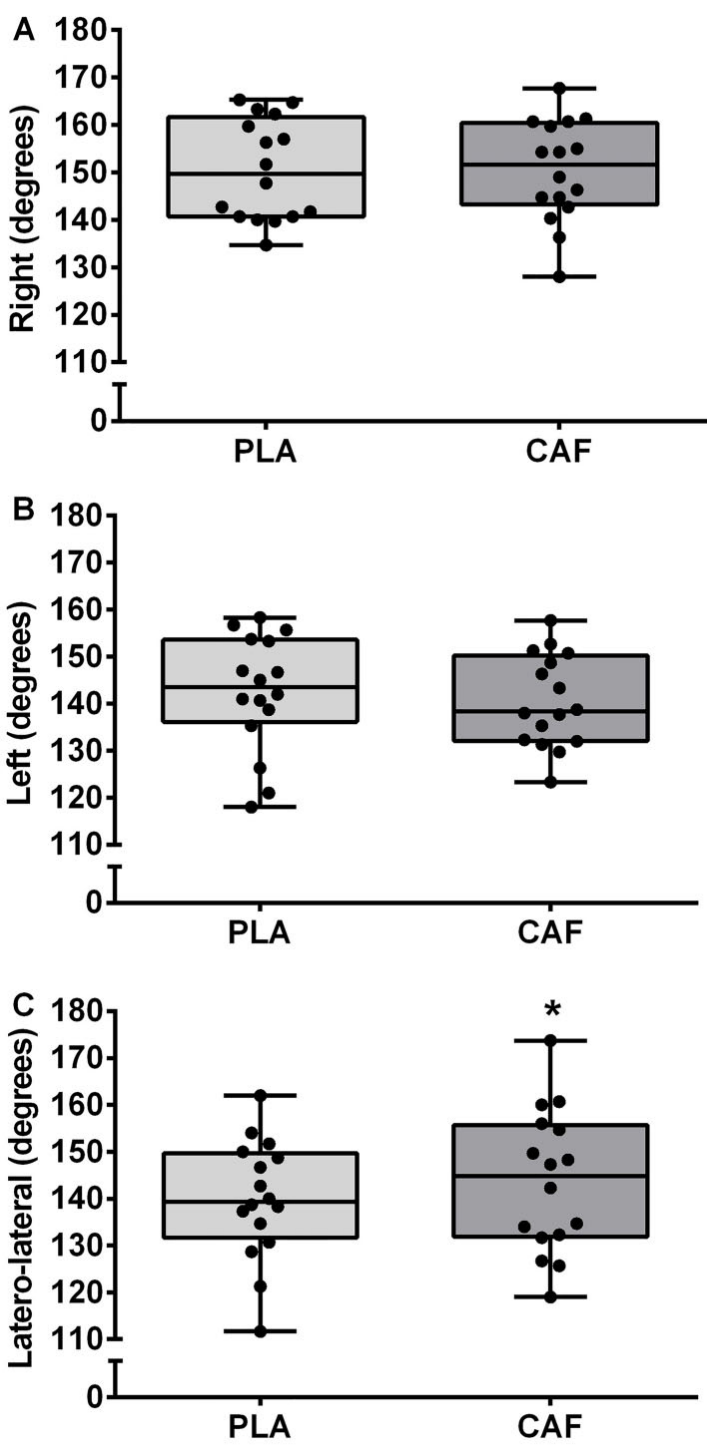
Amplitude of the Ariana figure after placebo (PLA) or caffeine (CAF) ingestion. Data are reported as means±SD. *P<0.05 compared to placebo (paired *t*-test).

Performance during the Ariana figure is shown in Figure 2. The right and left anteroposterior splits were not affected by caffeine ingestion (150.4±10.7 and 140.6±9.8 degrees), compared to the placebo (150.5±10.7 and 142.5±12.4 degrees, *t*(15)=0.09 and 0.89, P=0.937 and 0.387, and ES=0.01 [95%CI: -0.33, 0.35] and 0.16 [95%CI: -0.21, 0.54], trivial, respectively). The corresponding Bayes factors were 0.42 and 0.28 (anecdotal and substantial evidence in favor of the null hypothesis, respectively). However, caffeine increased latero-lateral amplitude (143.5±15.2 degrees) compared to the placebo (139.8±12.8 degrees, *t*(15)=-2.32, P=0.035, ES=0.25 [95%CI: 0.02, 0.50], small). The Bayes factor was 1.80 (anecdotal evidence in favor of the alternative hypothesis).

### Boost and Barracuda

**Figure 3:**
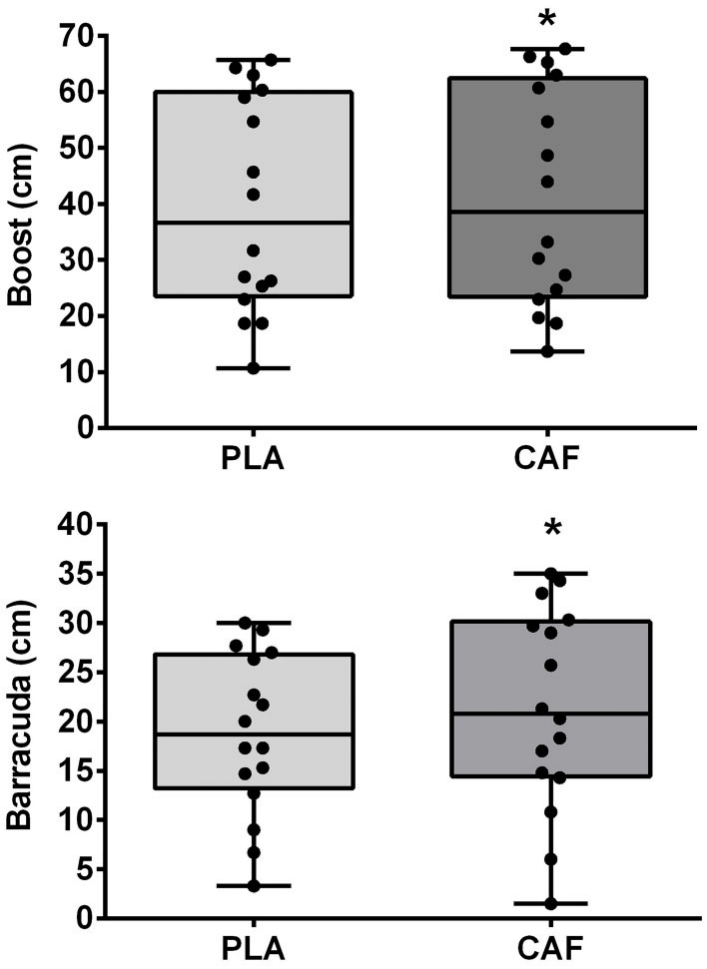
Maximal height reached during the Boost and Barracuda figures after placebo (PLA) or caffeine (CAF) ingestion. Data are reported as means±SD. *P<0.05 compared to placebo (paired *t*-test).

Performance during the Boost and Barracuda are shown in Figure 3. The height of the Boost was increased with caffeine (41.3±19.5 cm) compared to the placebo (39.7±19.2 cm, *t(15)*=-2.43, P=0.028, ES=0.08 [95%CI: 0.01, 0.15], trivial). Similarly, the height of the Barracuda was increased with caffeine (21.3±10.3 cm) compared to the placebo (18.8±8.2 cm, *t(15)*=-2.96, P=0.009, ES=0.25 [95%CI: 0.06, 0.47], small). The corresponding Bayes factors were 2.06 and 5.21 (anecdotal and substantial evidence in favor of the alternative hypothesis, respectively).

### Stationary Scull

**Figure 4:**
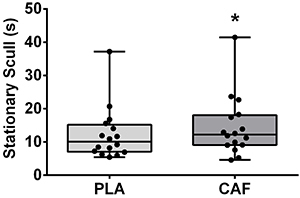
Time maintained during the Stationary Scull figure after placebo (PLA) or caffeine (CAF) ingestion. Data are reported means±SD. *P<0.05 compared to placebo (paired *t*-test).

Performance during the Stationary Scull is shown in Figure 4. Time supported at the required position was significantly longer with caffeine (14.5±9.1 s) compared to the placebo (12.3±7.9 s, *t(15)*=-2.84, P=0.012, ES=0.24 [95%CI: 0.05, 0.45], small). The Bayes factor was 4.24 (substantial evidence in favor of the alternative hypothesis).

### Order effect

There was no order effect for right and left anteroposterior and latero-lateral amplitudes during the Ariana (trial 1: 151.8±9.0, 143.3±11.2, and 141.0±12.9 degrees *vs* trial 2: 149.1±12.0, 139.8±11.0, and 142.3±15.3 degrees, respectively, all P>0.05). Similarly, there was no order effect (all P>0.05) for the Boost (trial 1: 39.9±19.3 *vs* trial 2: 41.1±19.4 cm), Barracuda (trial 1: 20.3±9.6 *vs* trial 2: 19.9±9.2 cm), and Stationary Scull (trial 1: 13.5±9.1 *vs* trial 2: 13.3±8.1 s).

## Discussion

This is the first study investigating the effect of caffeine on performance of movements of artistic swimming, which demanded flexibility (Ariana), strength and power (Boost and Barracuda), and muscular endurance (Stationary Scull). We found that ingesting 5 mg/kg of caffeine one hour before the tasks improved latero-lateral amplitude in the Ariana, increased maximal height during the Boost and Barracuda, and prolonged the time until task failure during the Stationary Scull. These findings indicated that caffeine could be considered as a supplement to improve the performance of artistic swimmers.

The right and left anteroposterior splits during Ariana were not affected by caffeine ingestion, but caffeine increased the latero-lateral amplitude compared to the placebo. During the execution of the Ariana, the better an athlete maintains the height and extension of the split position, close to 180°, throughout the execution, the better will be the score. The latero-lateral split is a symmetric movement that requires less stability of the trunk, which easily enables a greater joint amplitude. On the other hand, the anteroposterior split is an asymmetric movement of higher complexity, which requires greater stability of the trunk and the recruitment of additional muscles. Although speculative, a given acute intervention, as a supplement ingestion, may be more effective during movements demanding less complexity (i.e., latero-lateral split). More complex movements (i.e., anteroposterior split) may demand a higher level of processing, increasing the complexity of the intrinsic dynamics of the motor system and the stability of the movement pattern, which could reduce the action of an acute supplementation. We reinforce, however, that these assumptions are speculative and will demand further studies for confirmation. Nonetheless, although several different factors are analyzed by judges during the execution of the Ariana such as grace, artistry, and stability and accuracy of positions and transitions, excellent flexibility is essential to perform this movement well. Thus, caffeine-induced increases in flexibility might help athletes to attain a higher score during the execution of the Ariana or any other movement demanding increased flexibility (e.g., Barracuda Airborne Split, in which athletes must rapidly perform a dynamic leg split). It should be taken into account, however, that Bayes factor indicated an anecdotal evidence in favor of the alternative hypothesis. Thus, whether such small differences between caffeine and placebo would be perceptive and influence a judge's assessment are unclear. Further studies should advance by comparing scores attributed by blinded judges when athletes are performing the Ariana with and without caffeine intake.

Although there were small differences, our findings suggested that caffeine may have a positive effect on leg flexibility. The effect of caffeine on flexibility is underexplored and the mechanism by which caffeine might improve flexibility is not understood. To our knowledge, only one preliminary study from our laboratory investigated the effect of caffeine on flexibility, demonstrating that caffeine improves right and left forward splits measured on the ground (19). This study did not explore the mechanism by which caffeine improves flexibility, but it is known that caffeine reduces the perception of pain (18). Pain may be the main limiting factor of muscle stretch (31 [Bibr B32]); therefore, any reduction in pain perception might result in improved flexibility. In addition, because the Ariana demands active flexibility, caffeine-induced increases in force and power of thigh abductors will also contribute to larger latero-lateral amplitude. Further studies will be necessary to determine the mechanism for the caffeine-induced increase in flexibility.

In the present study, caffeine also increased the maximal height reached during the Boost and the Barracuda. The Bayes factor indicated, however, that the effects of caffeine were more pronounced in the Barracuda than in the Boost. Caffeine might increase muscle force and power by combining central and peripheral effects (11,32). A recent meta-analysis suggests that caffeine ingestion improves both muscle strength and power (24). It is interesting to note that a subgroup analysis indicated that caffeine significantly improves upper but not lower body strength (24). Our findings of a larger caffeine-induced improvement in the Barracuda compared to the Boost, where the performance in the former might be more dependent on arm strength than in the latter, are in accordance with this assumption. However, there is some data suggesting that caffeine induces an increase in strength primarily in knee extensors but not in the forearms (33). It is argued that larger muscles have greater motor unit recruitment capability with caffeine intake than smaller muscles (34). Our findings suggest, therefore, that caffeine ingestion is useful to improve muscle strength and power during exercises recruiting upper limbs such as the Barracuda, with a lower effect on exercises recruiting lower limbs such as the Boost.

The Barracuda is a compulsory figure in all categories of artistic swimming. The Boost is not compulsory, but it is usually present in a typical artistic routine. Reaching a high height in both movements is mandatory to attain a high score. Thus, caffeine-induced increases in muscle strength and power might be useful during figures or routine competitions containing the Barracuda and/or Boost, as well as during any other strength- or power-dependent movements (e.g., London figure, in which athletes must rapidly move from a horizontal to a vertical position). Nevertheless, as mentioned above, the impact of caffeine might be more pronounced during figures demanding force in the upper limbs.

The duration of the Stationary Scull was ∼18% longer after caffeine ingestion compared to the placebo, with Bayes factor indicating substantial evidence in favor of the alternative hypothesis (i.e., a positive effect of caffeine). Maintenance of the Stationary Scull is dependent on muscular endurance (2). Caffeine has been reported to increase muscular endurance, but it appears that caffeine does so only when it is assessed using open (∼18%) but not fixed endpoint tests (33). The Stationary Scull is an open-loop task in which athletes are requested to maintain the figure until failure. Thus, our findings reinforced that caffeine affects muscular endurance during open-loop tasks.

The Scull is not specifically evaluated during a typical routine or figure competition, but it is the most essential part of artistic swimming. Sculls serve to support or prepare for the next movement and are executed several times during a competition, demanding high muscular endurance. Thus, caffeine-induced increases in time for maintaining a Stationary Scull might help athletes to support the several transitions from a movement to another during a routine.

It is important to mention that swimmers compete individually during figure competition and then as a team during the routine (2). For figures, swimmers are ranked individually and performance during execution of each figure depends on flexibility, strength, power, and muscle endurance. Thus, our findings indicated a potential ergogenic effect of caffeine during figure competitions. Although we have not directly tested the effect of caffeine during a duo or team routine, and more elements are demanded during a duo/team presentation (e.g., teamwork and synchronization), caffeine-induced increases in flexibility, strength, power, and muscle endurance may help athletes to better perform difficult routines. This assumption is in agreement with several studies showing that caffeine has ergogenic effects in several sports demanding multiple physical capacities such as volleyball (35), basketball (36,37), and football (38).

A limitation of the present study is that performance during figure and/or routine competitions is not judged objectively for such things as the degrees of flexibility, strength, or power. Rather, performance is computed from the standpoint of perfection by a panel of judges attributing a score from zero to 10 using 1/10th-point scale (2). Thus, whether caffeine-induced improvement in the movements would be great enough to be converted into a higher score from judges deserves further investigation. However, although the magnitude of the effect of caffeine on specific artistic swimming tasks ranged from small to trivial, and Bayes factor indicated in some cases anecdotal evidence in favor of the alternative hypothesis, a small positive effect might be important and determine the score during a routine and/or figure competition at national and international levels. Our findings suggested, therefore, that coaches and athletes should consider the ingestion of caffeine before artistic swimming competitions. It is noteworthy that this is the first study investigating the effect of a given supplement on specific-artistic swimming tasks. Further studies should test other types of supplements that influence muscle strength and power (e.g., creatine monohydrate) on specific-artistic swimming tasks. In addition, due to the nature of our study, we were unable to explore the mechanist basis of the caffeine-induced improvements in performance, deserving further investigations. Finally, we have not determined the blinding effectiveness and, therefore, we cannot ascertain whether a potential supplement identification and participant's belief about caffeine being a potential ergogenic aid would influence exercise performance, as previously reported (39,40). The lack of a control condition also precludes the verification of a potential placebo effect (39,40). Further studies should measure the blinding effectiveness and include a control condition.

### Conclusions

In conclusion, caffeine ingestion increased performance during the execution of the Ariana, Boost, Barracuda, and Stationary Scull movements, all important movements of artistic swimming. However, by contrasting the likelihood of the data fitting under the null hypothesis with the likelihood of fitting under the alternative hypothesis (Bayes factor), our findings suggested that caffeine seemed to be relevant to improve performance during the execution of the Barracuda and Stationary Scull. Thus, caffeine seems to be a promising supplement to improve performance in some figures and elements of artistic swimming.
